# Cyclophilin A potentiates TRIM5α inhibition of HIV-1 nuclear import without promoting TRIM5α binding to the viral capsid

**DOI:** 10.1371/journal.pone.0182298

**Published:** 2017-08-02

**Authors:** Mallori Burse, Jiong Shi, Christopher Aiken

**Affiliations:** Department of Pathology, Immunology and Microbiology, Vanderbilt University Medical Center, Nashville, Tennessee, United States of America; Scripps Research Institute, UNITED STATES

## Abstract

The host immunophilin cyclophilin A (CypA) binds to the capsid protein (CA) of HIV-1 and regulates its infectivity. Depending on the target cell type, CypA can either promote or inhibit HIV-1 infection. The ability of CypA to promote HIV-1 infection has been extensively studied and linked to several steps in early replication including uncoating, reverse transcription and nuclear import. By contrast, the mechanism by which CypA inhibits infection is less well understood. We investigated the mechanism by which CypA potentiates restriction of HIV-1 by the tripartite motif-containing protein 5 (TRIM5α). Depletion of TRIM5α in the African green monkey cell line Vero, resulted in a loss of inhibition of infection by CypA, demonstrating that inhibition by CypA is mediated by TRIM5α. Complementary genetic and biochemical assays failed to demonstrate an ability of CypA to promote binding of TRIM5α to the viral capsid. TRIM5α inhibits HIV-1 reverse transcription in a proteasome-dependent manner; however, we observed that inhibition of proteasome activity did not reduce the ability of CypA to inhibit infection, suggesting that CypA acts at a step after reverse transcription. Accordingly, we observed a CypA-dependent reduction in the accumulation of nuclear HIV-1 DNA, indicating that CypA specifically promotes TRIM5α inhibition of HIV-1 nuclear import. We also observed that the ability of CypA to inhibit HIV-1 infection is abolished by amino acid substitutions within the conserved CPSF6-binding surface in CA. Our results indicate that CypA inhibits HIV-1 infection in Vero cells not by promoting TRIM5α binding to the capsid but by blocking nuclear import of the HIV-1 preintegration complex.

## Introduction

In the early stages of human immunodeficiency virus type I (HIV-1) infection, a subviral complex comprised of the capsid (CA) protein shell, the viral genome, and accessory proteins, known as the core, undergoes a controlled disassembly process termed uncoating [1–4]. HIV-1 uncoating is influenced by the sequence of HIV-1 CA, and facilitates reverse transcription of viral RNA and particle entry into the nucleus, where the virus gains access to host chromatin [4–6]. The role of CA during infection is typically considered in terms of this structural contribution as the delivery vehicle for the viral genome. However, HIV-1 CA plays an increasingly appreciated role in virus-host interactions, both with co-factors repurposed by the virus to support replication, and with restriction factors elaborated by the cell to prevent infection.

TRIM5α is a restriction factor that blocks retroviral infection by binding to the capsid [7–11]. TRIM5α restriction is species specific; for example African green monkey TRIM5α (TRIM5α_agm_) restricts infection by HIV-1, simian immunodeficiency virus from rhesus macaques (SIVmac) and N-tropic murine leukemia virus (N-MLV). By contrast, the human TRIM5α restricts infection by N-MLV but not the closely related B-tropic MLV, or HIV-1. TRIM5α restriction of HIV-1 is initiated by binding of TRIM5α to the virus via an array of low affinity binding sites on the surface of the viral capsid. TRIM5α accelerates uncoating and potently blocks HIV-1 reverse transcription [7,12]. Biochemical inhibition of the proteasome, or specific mutations to the RING domain uncouple the block to reverse transcription from inhibition of infection, demonstrating that TRIM5α can also inhibit steps after reverse transcription [13,14].The TRIM protein family is characterized by the presence of the tripartite RBCC motif, consisting of a RING domain, one or more B-box domains, and a coiled-coil domain. In addition to its canonical amino-terminal RBCC motif, TRIM5α also contains a SPRY (B30.2) domain at its carboxy-terminal end that facilitates species specific recognition of retroviral capsids [7,8,15–17]. In addition to restricting infection, TRIM proteins have been implicated in innate immune signaling, suggesting an general role for these proteins in host defense [18,19].

The host protein cyclophilin A (CypA) enhances the ability of TRIM5α to block HIV-1 infection [20–22]. CypA is a peptidyl-prolyl isomerase that binds to the N-terminal domain of HIV-1 CA, making contacts with glycine 89 and proline 90 [23]. Mutations to either of these residues, or treatment with the immunosuppressive drug cyclosporine A (CsA), inhibit CypA binding to CA [24]. The precise role for CypA during infection has remained somewhat elusive due to its paradoxical ability to either enhance or inhibit HIV-1 infection, depending on the cell type. The positive effects of CypA have been linked to its ability to stabilize the capsid, alter uncoating and increase the efficiency of reverse transcription and nuclear import [25–28]. CypA regulates the dependence of HIV-1 on several nuclear-pore proteins, which are thought to support the ability of HIV-1 to traverse the nuclear pore, and to influence integration site preferences [28–30]. The ability of CypA to inhibit HIV-1 infection is less well understood, but seems to be related to the function of inhibitory capsid-binding molecules. In addition to TRIM5α, CypA-dependence has also been demonstrated for myxovirus resistance protein B (MxB), an interferon-inducible protein that inhibits HIV-1 nuclear import, and for the small molecule inhibitor PF74, which accelerates HIV-1 uncoating [31–33]. CypA-dependent inhibition also occurs during infection of cell-cycle dependent HIV-1 CA mutants, an effect that requires CPSF6. [34,35].

The mechanism by which CypA potentiates TRIM5α restriction of HIV-1 is poorly understood. Initial observations showed that depletion of CypA, or treatment of cells with CsA, promotes HIV-1 infection of cells expressing old world monkey orthologues of TRIM5α. Infection of such cells by HIV-1 CA mutants that do not bind CypA, such as G89V and P90A, is not enhanced by CsA, indicating that CypA mediates its effect through its binding to the HIV-1 capsid [20,36]. Multiple alleles of TRIM5α including TRIM5α_agm_ and TRIM5α from rhesus macaques (TRIM5α_rh_), exhibit stronger restriction of HIV-1 in the presence of CypA. However, only HIV-1 is subject to CypA-dependent restriction, despite the ability of other retroviruses such as SIV from tantalus monkeys (SIV_agmtan_) to bind CypA [36,37]. In this study, we sought to understand the mechanism by which CypA promotes HIV-1 restriction by TRIM5α.

## Materials and methods

### Cells and viruses

Vero and Owl Monkey Kidney (OMK) cell lines were purchased from the American Type Culture Collection (ATCC cat. nos. CCL-81 and CRL-1556, respectively). The 293T-pLPCX-TRIM5α_agm_ cell line was previously described [38]. All cells were cultured at 37°C in 5% CO_2_ in Dulbecco's modified Eagle's medium (DMEM) supplemented with fetal bovine serum (10%), penicillin (100 U/ml), and streptomycin (100 μg/ml) (D10). Virus stocks were generated by calcium chloride transfection of 293T cells for infectivity assays, or with TransIT-293 Transfection Reagent (Mirus) for use in experiments involving real-time PCR [39]. HIV-1 reporter virus was produced from HIV-GFP, an Env-defective HIV-1 proviral construct encoding green fluorescent protein (GFP) in place of Nef [40], or from the corresponding mutants encoding a substitution for proline at position 90 (P90A), for asparagine at postion 74 (N74D), for alanine at position 105 (A105T), and for threonine at position 107 (T107N). Particles were pseudotyped by vesicular stomatitis virus glycoprotein (VSV-G) via cotransfection of the proviral constructs (20 μg) with pHCMV-G (5 μg). VSV-G-pseudotyped HIV-1 particles used in abrogation-of-restriction assays were produced from Env-defective R9 (R9ΔE) proviral construct and the corresponding P90A mutant proviral constructs. HIV-1 stocks were quantified using a p24 enzyme-linked immunosorbent assay (ELISA) or titrated on CRFK cells to normalize infectious dose, aliquoted and stored at -80°C prior to use. N-tropic murine leukemia virus (N-MLV) and B-tropic MLV vector particles were produced by cotransfection of pVPack-GP-N (Agilent Technologies) or pVPack-GP-B (encoding N-tropic or B-tropic MLV Gag-Pol respectively) (20 μg) with pBABE-EGFP (15 μg) and pHCMV-G (5 μg).

### shRNA-mediated depletion

Lentiviral vectors expressing Mission short hairpin RNA against each TRIM5α (Sigma, no. TRCN0000005897; hairpin sequence, CCGGGCACTGTCTCATTCTT-CAATACTCGAGTATTGAAGAATGAGACAGTGCTTTTT), CypA (Sigma, no. TRCN0000309687; hairpin sequence, CCGGCATCAAACCATTCCTTCTGTACT-CGAGTACAGAAGGAATGGTTTGATGTTTTTG), or Mission pLKO.1-puro empty (SHC001) were prepared by calcium chloride transfection of 293T cells. pLKO.1 (20ug) was cotransfected with psPAXII (15 μg) and pHCMV-G (5 μg) to produce VSV-G pseudotyped particles [41]. Subconfluent cultures of Vero cells were then inoculated with each of these vectors; 48 h later, the transduced cells were selected in puromycin (10 μg/ml) for an additional 48 h. Depletion was confirmed by harvesting mRNA from puromycin-resistant cell populations using an RNeasy RNA Isolation kit (Qiagen). Isolated RNA was subject to cDNA synthesis using iScript cDNA synthesis kit (Life Technologies). cDNA transcripts were quantified by real-time PCR using SYBR green for detection with a Stratagene MX3000p instrument. Primers used to detect TRIM5α and CypA mRNA were T5AFWD 5’-GGTCATTTGCTGGCTTTGTG-3’ T5AREV 5’-TCGTAGTCTATTTGAATCTTCCAGG-3’ and CypAFWD 5’- CCAGGGTTTATGTGTCAGGG-3’ CypAREV 5’- CCATCCAACCACTCAGTCTTG-3’ respectively. GAPDH mRNA was quantified using GAPFWD 5’-ATGACATCAAGAAGGTGGTG-3’ and GAPREV 5’-CATACCAGGAAATGAGCTTG-3’ to normalize quantification of target mRNAs. TRIM5α or CypA mean threshold cycle (Ct) values were normalized to the Ct value for GAPDH of each sample using the ΔΔCt method.

### Infection assays

Vero cells (15,000 per well) were seeded in a 96-well plate. The next day, cells were inoculated with GFP-encoding HIV-1 for 16 h in the presence of polybrene (8 μg/ml) and cyclosporine A (CsA, Calbiochem) (5 μM) where indicated. 16 h after virus inoculation, virus and drugs were removed and replaced with fresh media. 48 h after infection, cells were detached with trypsin-EDTA and fixed with an equal volume of phosphate-buffered saline (PBS) containing 4% paraformaldehyde. The extent of infection was assayed by flow cytometry for GFP expression using an Accuri C6 flow cytometer. A minimum of 5,000 cells was analyzed for each sample.

### RNA sequencing and transcriptome analysis

Total RNA was isolated in triplicate from 60% confluent, 6 cm dishes of Vero or OMK cells using the RNeasy RNA Isolation kit (Qiagen) according to manufacturer’s instructions. The quality of RNA was assessed by capillary electrophoresis using the Bioanalyzer 2100 (Agilent Genomics). Samples that received a RNA integrity (RIN) score lower than 8 were discarded. Total RNA libraries were prepared for each sample using the TruSeq Stranded Total RNA kit (Illumina) and subject to Illumina HiSeq PE75 sequencing. The resulting sequencing reads were imported into CLC Genomics Workbench 10 (**https://www.qiagenbioinformatics.com/**) as paired-end reads and analyzed by two workflows 1) de novo assembly and BLAST with the following parameters automatic word size: 45, bubble size: 98, minimum contig length: 1000 and read mapping back to contigs or 2) read mapping to TRIMCyp sequences. Assembled contig sequences resulting from de novo assembly (workflow 1) were pooled to create BLAST databases for Vero and OMK samples using CLC Genomics ‘Create BLAST database’ feature. BLAST databases were queried for contigs aligning to OMK TRIMCyp, TRIM5, or CypA. BLAST Parameters were as follows, Match cost 1, Mismatch cost 1, Gap Existence 2, Gap Extension 2, Expectation value = 10.0, Word size = 11, Mask lower case = No, Filter low complexity = Yes, Maximum number of hits = 250, Number of threads = 4. To assess read alignment in the TRIM and CypA junction region of TRIMCyp we mapped each read library to OMK TRIMCyp using CLC Genomics WorkBench ‘Map Reads to Reference’ Tool. Coverage analysis of junction regions were assessed using the ‘Coverage Analysis’ Tool.

The complete high throughput sequencing dataset has been submitted to the Sequence Read Archive (SRA) (http://www.ncbi.nlm.nih.gov/sra) under accession number PRJNA388683.

### *In vitro* binding assay

Extracts from 293T cells stably expressing African green monkey TRIM5α tagged with hemagglutinin epitope were prepared by hypotonic lysis; recombinant CA protein was purified by anion exchange chromatography as previously described [35]. Crosslinked recombinant CA assemblies (5μM, 12.5 μg) were incubated with cell extracts (125–500 μg) for 1 h with inversion mixing every 15 min at room temperature in 100μl reactions in binding buffer (50 mM Tris pH 8.0, 150 mM NaCl, 5 mM MgCl_2_, and 0.5 mM EDTA) as previously described [35]. When indicated, CsA was included at 5 μM concentration. CypA was prepared as previously described, and was included at 8, 16, or 32 μM concentrations as indicated [25]. Reactions were then centrifuged for 5 min at 5,000x*g*, and the pellets rinsed with 100 μl of binding buffer before a second centrifugation for 5 min at 5,000x*g*. The pellets were analyzed by SDS-PAGE and immunoblotting with antibodies specific for CA (183-H12-5C) [42], CypA (Millipore), and HA epitope (Roche, 100 ng/ml) to detect TRIM5α. Band density was quantified using Licor Image Studio with the top and bottom background subtraction. TRIM5α-HA binding was determined by calculating the ratio of the TRIM5α-HA band intensity to that of CA.

### Abrogation-of-restriction assay

The abrogation-of-restriction assay was performed as previously described [43]. VSV-G pseudotyped N-MLV-GFP and B-MLV GFP reporter particles were titrated onto Vero cell monolayers to determine the dose corresponding to approximately 1% infection. Vero cells (15,000 cells per well in 96-well plates) were inoculated with a fixed quantity of N-MLV GFP or B-MLV GFP virus together with titrating concentrations of VSV-G-pseudotyped HIV-1 particles in the presence of polybrene (8 μg/ml). 16 h after inoculation, virus and media were removed and replaced with media. 48 h after inoculation, cells were detached with trypsin-EDTA and fixed with an equal volume of PBS containing 4% paraformaldehyde. Extent of infection was quantified by flow cytometry with an Accuri C6 flow cytometer. At least 5,000 cells were analyzed for each sample.

### Real-time PCR to detect HIV-1 reverse transcripts

Vero cell cultures were inoculated with DNase I-treated stocks of Env-defective HIV-1 particles pseudotyped with VSV-G in the presence or absence of CsA (5 μM), MG132 (5μM), both, or Efavirenz (1 μM) as indicated. 12h post infection, cells were detached with trypsin-EDTA, pelleted, and washed once with 1X PBS. The cells were then treated with lysis buffer (50mM KCl, 1.5mM MgCl_2,_ 1.5mM Tris-HCl pH 8.0, 0.45% NP40 and 0.45% Tween-20) containing proteinase K (1 mg/ml) for 1 hr at 57°C. To inactivate proteinase K, samples were incubated at 95°C for 15 minutes then stored at -80°C until further use. HIV-1 DNA in the samples was quantified by real-time PCR using primers specific for second strand transfer products LateRT-FWD 5′-AGCAGCTGCTTTTTGCCTGTACT-3′ and LateRT-Rev 5′-CCTGCGTCGAGAGATCTCCTCTGG-3′ and 2-LTR circle products were detected with J2 5′–C AGTGTGGAAAATCTCTAGCAGTAC–3′ and JRev 5′–GCCGTGCGCGCTTCAGCAAGC–3′ using SYBR green detection in a Stratagene MX3000p instrument. GAPDH was also quantified in each sample to normalize HIV-1 cycle threshold (C(t)) values by GAPDH C(t) values to generate delta delta Ct (ΔΔCt) values.

## Results

### Cyclophilin A interaction with the HIV-1 capsid potentiates TRIM5α restriction

**Fig 1:**
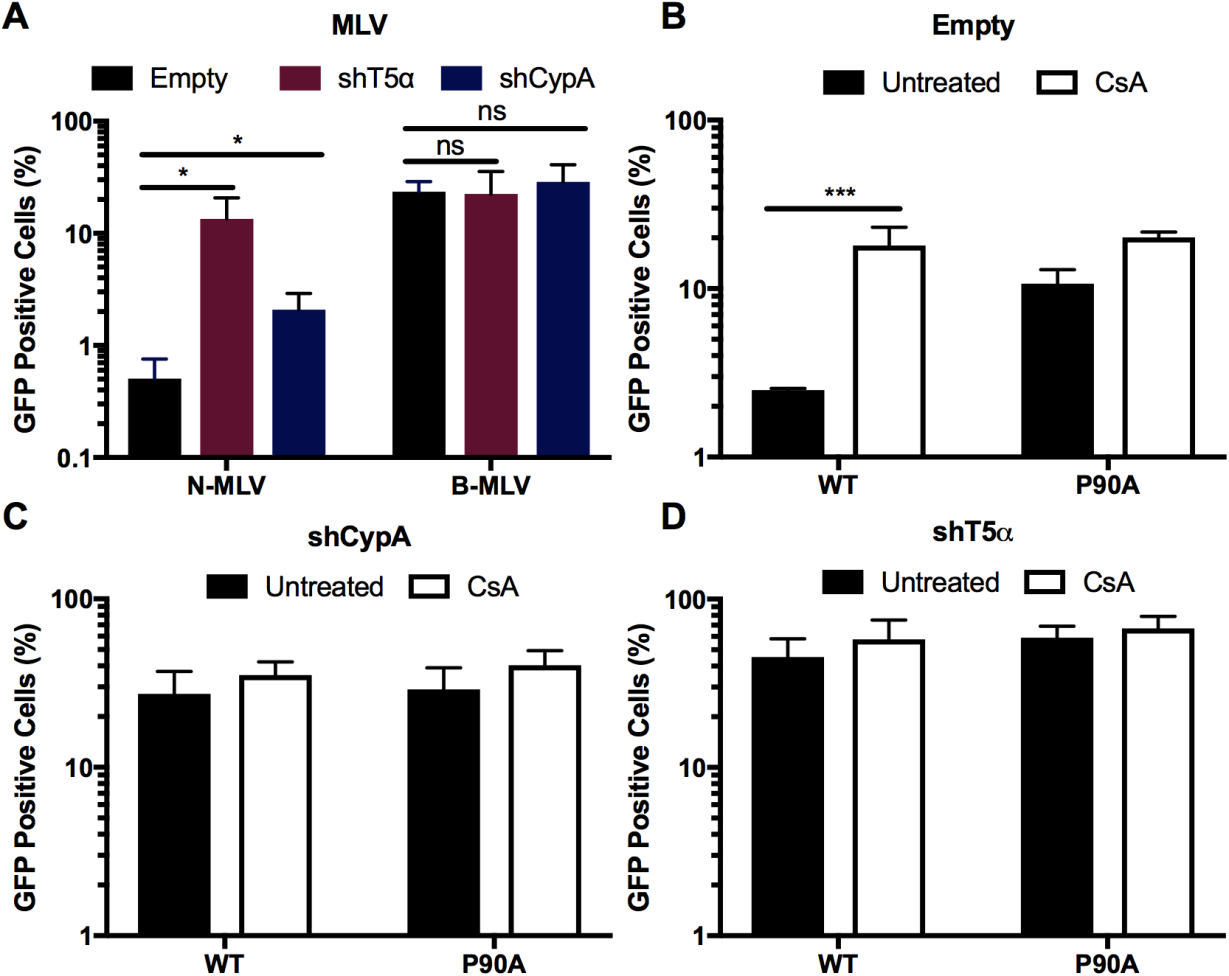
CypA potentiates TRIM5α restriction of HIV-1. (A) Vero cells depleted of TRIM5α (shT5α) or CypA (shCypA), and control (Empty) were assayed for infection by the indicated MLV or HIV-1 reporter viruses (B-D), in the presence and absence of CsA. Results shown are the mean values from 3 independent experiments, error bars reflect the standard error of the mean, Unpaired t-test, * = p<0.05, ** = p<0.005, *** = p<0.0005.

To confirm the previously reported ability of CypA to potentiate TRIM5α restriction in old world monkey cells, we examined the effect of inhibiting CypA-CA interactions in Vero cells [20,21,36,44]. Vero cells were stably depleted of TRIM5α (shT5α) or CypA (shCypA) using shRNAs directed against the corresponding mRNAs. As a control, cells were transduced with the corresponding vector lacking an shRNA (shEmpty). Depletion of the mRNAs was confirmed by quantitative RT-PCR (S1 Fig). To assess functional depletion of proteins, we used N-tropic (N-MLV) and B-tropic MLV (B-MLV). N-MLV is potently restricted by TRIM5α while B-MLV is not, and neither virus binds CypA [45,46]. As expected, depletion of TRIM5α markedly enhanced N-MLV infection, while B-MLV infectivity was unaffected by depletion of either protein (Fig 1A). Depletion of CypA moderately increased N-MLV infection (Fig 1A). We then inoculated the Vero cell lines with a GFP-encoding HIV-1 reporter virus pseudotyped with VSV-G in the presence and absence of CsA. The non-CypA binding mutant P90A HIV-GFP was included as a negative control. As previously reported, CsA treatment or CypA depletion markedly increased wildtype but not P90A HIV-1 infection (Fig 1B and 1C, [20,21,36]). Depletion of TRIM5α also increased infection by wild type HIV-1, and markedly reduced the enhancement of infection by CsA (Fig 1D). P90A infectivity was enhanced less than 2-fold by CsA treatment in control and TRIM5α-depleted cells (Fig 1B and 1D). CypA depletion also increased infectivity of P90A HIV-1 although to a much less extent than wild type HIV-1 (Fig 1C). These results indicate that binding of CypA to the incoming viral capsid inhibits HIV-1 infection of Vero cells by a mechanism that depends on expression of TRIM5α.

### Transcriptome mining for TRIMCyp in Vero cells

**Fig 2:**
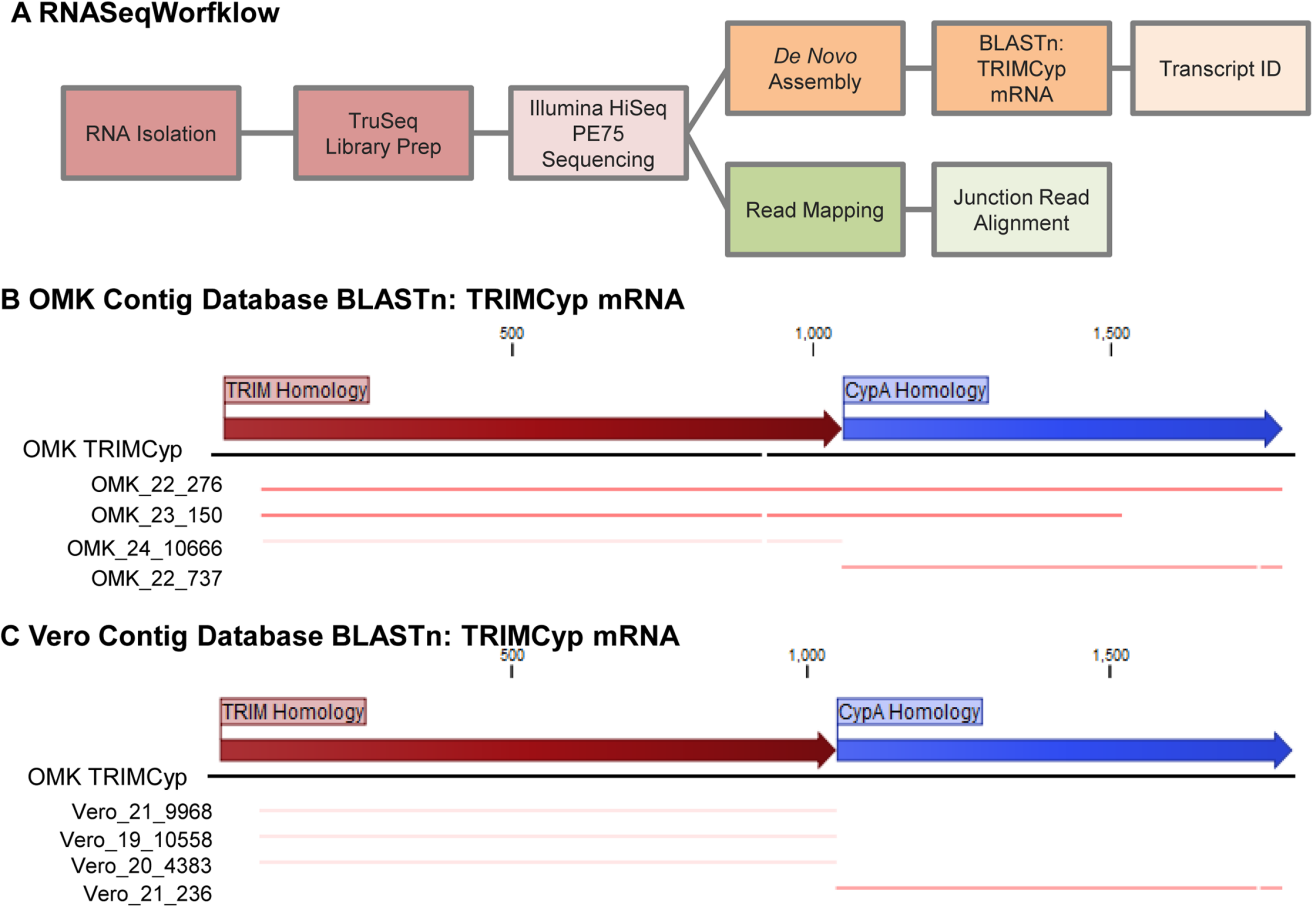
TRIMCyp transcripts were not detected in Vero cells by RNASeq. (A) RNASeq workflow. Total RNA was isolated from triplicate samples of Vero and OMK cells and subject to Illumina HiSeq PE75 sequencing. Sequencing reads were assembled using the CLC Genomics Workbench 10 *de novo* assembler. The resulting contigs were used to create BLAST databases to assess the presence of TRIMCyp transcripts. Alignment of top BLAST hits for OMK (B) or Vero (C) to TRIMCyp sequence.

TRIMCyp is a closely related family member of TRIM5α [47]. TRIMCyp, which consists of the RBCC domains of TRIM5α, but has CypA in place of the SPRY domain, was first identified in an owl monkey kidney cell line (OMK) but was later detected in some rhesus monkey subspecies, suggesting that TRIMCyp genes could exist in a variety of old world monkey cell lines [48–50]. Restriction by TRIMCyp occurs via binding of the CypA domain of the protein to the viral capsid, and is prevented by CsA treatment. Therefore, we considered the possibility that the apparent ability of CypA to promote restriction of HIV-1 in Vero cells might be a consequence of expression of a previously unreported TRIMCyp protein. To determine whether a TRIMCyp mRNA is present in Vero cells, we analyzed the transcriptome of Vero cells by RNAseq. OMK cells were included as a positive control for the presence and recovery of TRIMCyp transcripts. Total RNA was isolated from Vero and OMK cells in triplicate and subjected to Illumina HiSeq sequencing, which yielded an average of 68.3 million reads for OMK samples and 73.6 million reads for Vero samples (S1 Table). These reads were subject to *de novo* assembly using CLC Genomics Workbench 10. The contigs, continuous sequences resulting overlapping reads, generated from assembly of the triplicate Vero and OMK samples were pooled to create BLAST databases for each species. These databases were queried for contigs that aligned to *Aotus trivirgartus* TRIMCyp mRNA (OMK TRIMCyp) (Fig 2A, and summarized in S1–S3 Tables). Alignment of the top 4 BLAST hits from Vero and OMK contig databases are shown in Fig 2B and 2C respectively. Two OMK contigs, OMK_contig_150 and OMK_contig_276 respectively, aligned to TRIMCyp regions that span the junction between TRIM and CypA homology regions, indicating that TRIMCyp transcripts could be detected and assembled using the CLC Genomics *de novo* assembler algorithm. However, none of the Vero contigs aligned with the junction between TRIM5 and CypA homology regions, suggesting that a fusion transcript is not present in the transcriptome of Vero cells. We also generated two BLAST libraries for each Vero and OMK species that contained contigs which aligned to either CypA or TRIM5α. The TRIM5 libraries were then queried for CypA alignment, and the CypA libraries were queried for TRIM5 alignment. As outlined in S2 Table only two sequences scored as hits in these searches. The previously identified OMK_contig_150 and OMK_contig_276 aligned with both TRIM5 and CypA queries, however no sequences with alignment to both TRIM5 and CypA were identified from the Vero BLAST libraries. Finally, we assessed read coverage within the TRIM-Cyp junction by mapping the read libraries directly to OMK TRIMCyp. Although the read coverage in the junction region was similar between Vero and OMK samples, there were qualitative differences (S2 Fig), such that the Vero reads primarily aligned to either side of the TRIM-CypA junction but did not span it, while the OMK reads mapped across the junction. Together, these results indicate that Vero cells do not express a TRIMCyp protein and that the inhibition of HIV-1 infection by CypA does not result from TRIMCyp restriction.

### CypA-CA interactions do not promote TRIM5α binding to the capsid

**Fig 3:**
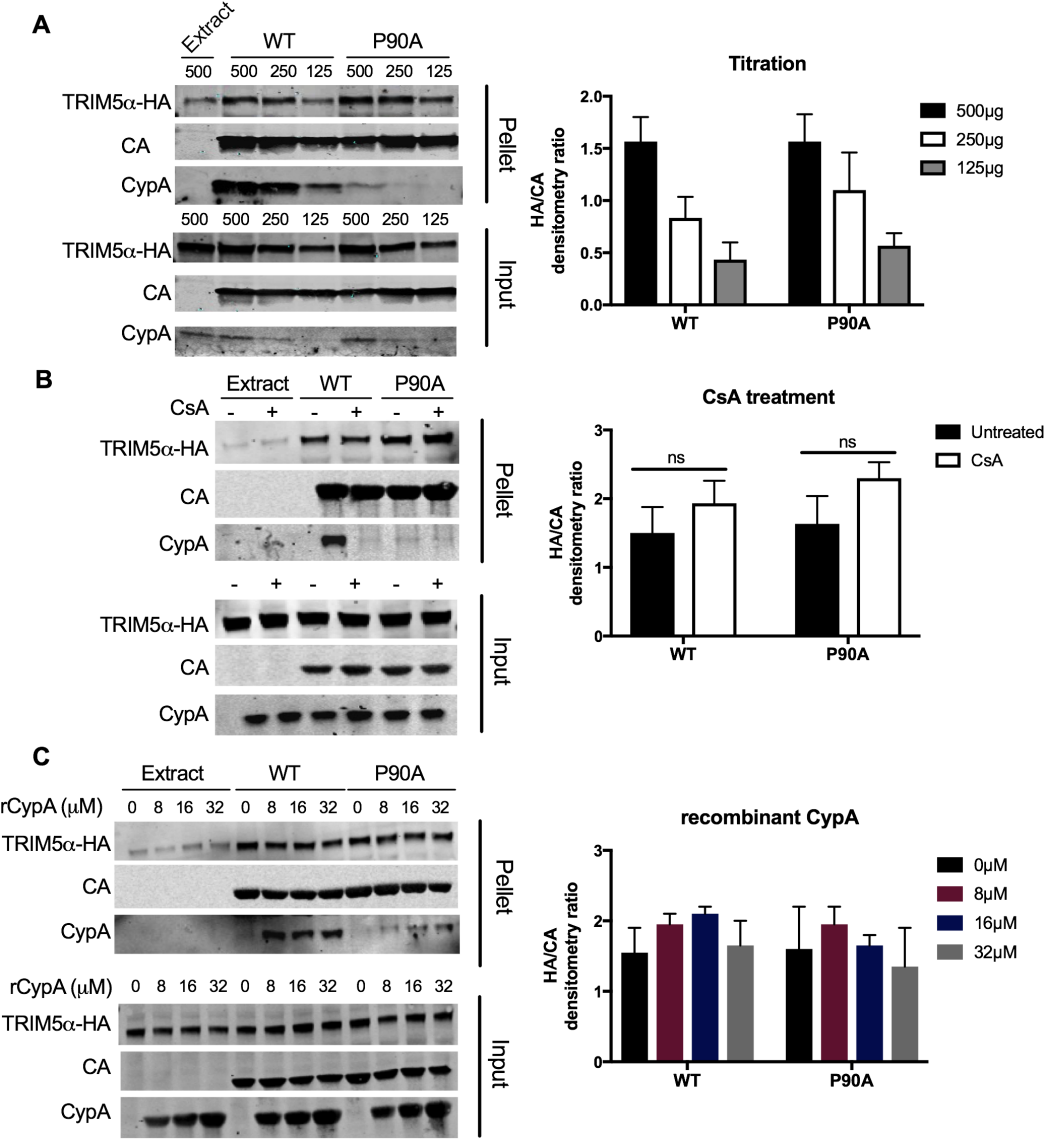
CypA does not enhance TRIM5α association with recombinant CA tubes. Recombinant CA tubular assemblies were incubated with extracts from 293T cells expressing TRIM5α-HA. CA tubes and associated proteins were pelleted and analyzed by SDS-PAGE and immunoblotting for HA, CA and CypA. Graphs shown to the right of each blot represent the quantification of blots. Data shown are the mean values of three independent determinations; error bars represent the standard error of the mean. (A) WT or P90A CA tubes were incubated with the indicated quantities of TRIM5α-HA cell extracts. (B) WT or P90A tubes were incubated with TRIM5α-HA cell extract (250 μg protein) in the presence or absence of CsA. (C) WT or P90A tubes were incubated with 250 μg TRIM5α-HA extracts and the indicated added concentrations of recombinant CypA in the presence or absence of CsA. Results shown are the mean values from 3 independent experiments, error bars reflect the standard error of the mean. Unpaired t-test, ns = not significant.

TRIM5α must bind to the incoming viral capsid to inhibit infection. Previous studies demonstrated that CypA acts shortly after virus fusion to promote HIV-1 restriction by TRIM5α [36]. Together with the well-known ability of CypA to isomerize the Gly89-Pro90 bond of HIV-1 CA and to alter its dynamics, we hypothesized that CypA enhances TRIM5α binding to the capsid in order to potentiate restriction. To test this hypothesis, we assayed TRIM5α binding to recombinant tubular assemblies of CA that mimic the hexagonal lattice structure of the capsid. Extracts were generated from 293T cells ectopically expressing hemagluttinin-tagged African green monkey TRIM5α (TRIM5α-HA). Disulfide-stabilized tubular CA assemblies were formed by the assembly of recombinant CA protein containing Cys substitutions at codons 14 and 45 (CA numbering). The CA tubes were incubated with cell extracts, and the complexes were pelleted by centrifugation. The pelleted complexes were subjected to SDS-PAGE and analyzed by immunoblotting for the HA-tagged TRIM5α protein. First, we compared the association of TRIM5α with WT CA tubes and with mutant P90A tubes, which are impaired for binding to CypA. We observed similar levels of TRIM5α-HA co-pelleting with WT and P90A tubes; however, with an intermediate quantity of cell extract, P90A bound slightly more TRIM5α-HA (Fig 3A). Next, we asked whether biochemical inhibition of CypA binding would alter the quantity of TRIM5α-HA bound to the tubes. Using the intermediate quantity of cell extract (250 μg), we performed binding assays in the presence and absence of CsA. CsA did not affect the level of TRIM5α-HA associated with the wild type or P90A tubes (Fig 3B). Probing the blot with a CypA-specific antibody confirmed that the CypA protein in the cell extract was bound to the wild type but not the P90A CA tubes, and that CsA prevented CypA association with the former. Thus, neither genetic nor biochemical inhibition of CypA binding to CA decreased TRIM5α-HA binding to CA tubes *in vitro*. The affinity of CypA for CA is approximately 16 μM [24]. Because the concentration of CypA in the cell extracts was necessarily lower than that in intact cells, we considered the possibility that the concentration of CypA present in the binding reactions is insufficient to promote the binding of TRIM5α to the capsid. To test this, we added recombinant CypA at concentrations of 8, 16, or 32 μM to the reactions and determined the effects on TRIM5α binding (Fig 3C). The addition of recombinant CypA did not increase the binding of TRIM5α at any CypA concentration tested. These *in vitro* data suggested that CypA does not enhance TRIM5α binding to the capsid.

**Fig 4:**
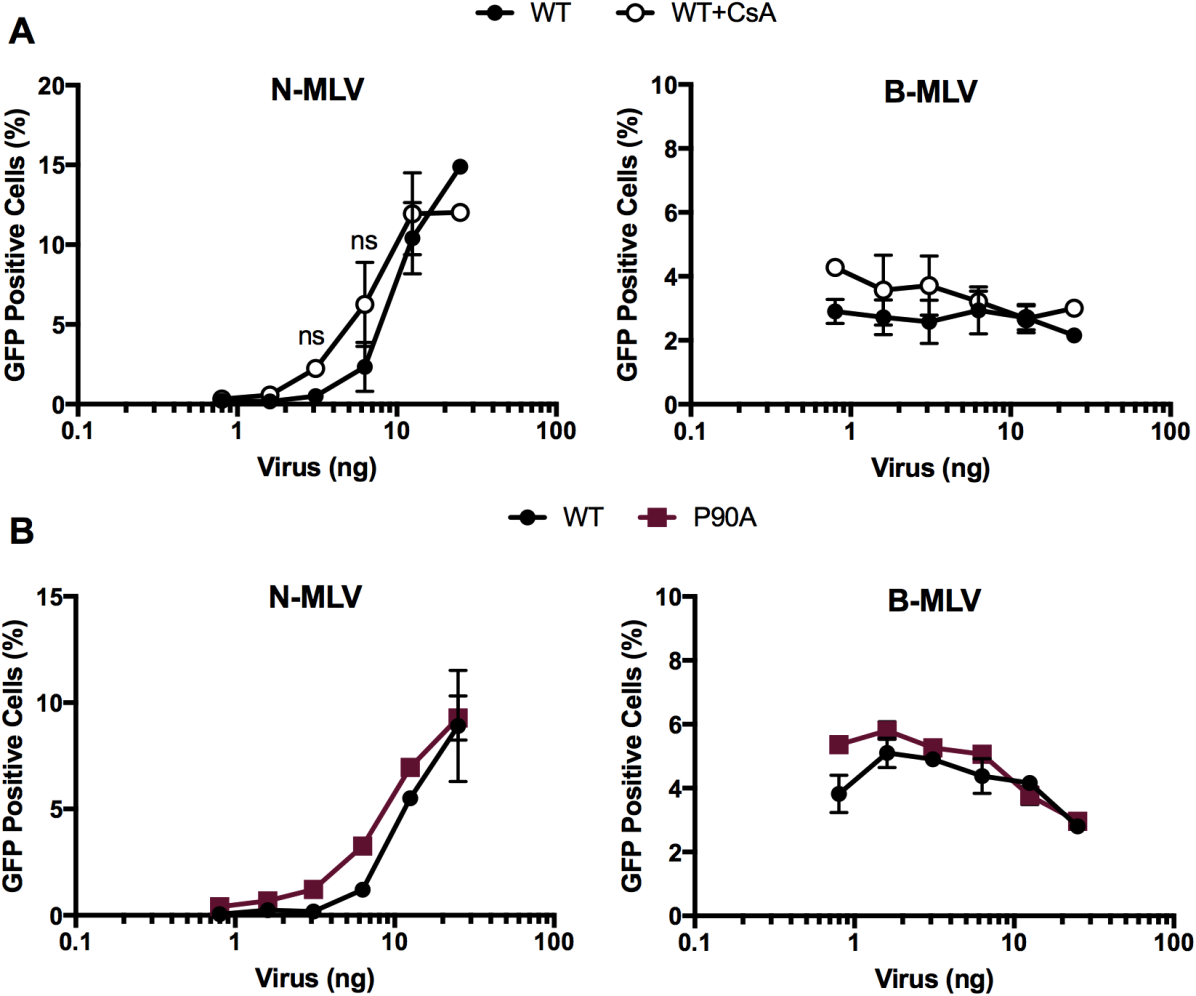
CypA-CA interactions do not enhance saturation of TRIM5α *in trans*. Vero cells were inoculated with a fixed dose of N-MLV or B-MLV GFP reporter viruses and titrating quantities of wild type HIV-1 in the presence and absence of CsA (A), or with the P90A mutant virus (B). Extent of infection was quantified by flow cytometry. Results shown are the mean values from 3 independent experiments; error bars reflect the standard error of the mean. One-way ANOVA, with Sidak’s multiple comparison test, ns = not significant.

To probe the effects of CypA on TRIM5α-capsid binding in cells, we exploited the ability of HIV-1 particles to overcome TRIM5α restriction *in trans* by saturation. The sequence and stability of the capsid dictate the ability of a virus to saturate TRIM5α restriction, making some viruses more efficient at saturation than others [43,51]. By comparing the ability of different viruses to saturate restriction *in trans*, one can infer differences in TRIM5α binding. We measured the infectivity of a fixed dose of GFP-encoding reporter virus when co-infected with increasing quantities of “decoy” particles. As reporter viruses, we employed N-MLV and B-MLV retroviruses to dissociate the effect of CypA on the abrogating virus from an effect on the reporter virus. B-MLV, which is not restricted by TRIM5α, was included as a negative control for loss of restriction at higher doses of abrogating virus. A fixed, sub-saturating dose of these viruses was inoculated together with increasing doses of wild type or P90A HIV-1 decoy particles. We also tested the ability of CsA to enhance wild type infection. Addition of wild type HIV-1 increased the infection by N-MLV from 0.5% to 15% infection, reflecting an ability to saturate restriction (Fig 4A); however the addition of CsA did not reduce the ability of HIV-1 to saturate restriction (Fig 4A). Similarly, P90A HIV-1 decoy particles abrogated restriction of N-MLV as effectively as wild type HIV-1 (Fig 4B). Infection by B-MLV particles was not enhanced by HIV-1 particles at any dose tested, consistent with the known selectivity of TRIM5α for restriction of N-MLV (Fig 4A and 4B, right-hand panels). Together with the *in vitro* binding results, these data indicate that CypA does not promote TRIM5α recognition of the HIV-1 capsid in target cells.

### Proteasome activity is dispensable for CypA inhibition of HIV-1 infection

**Fig 5:**
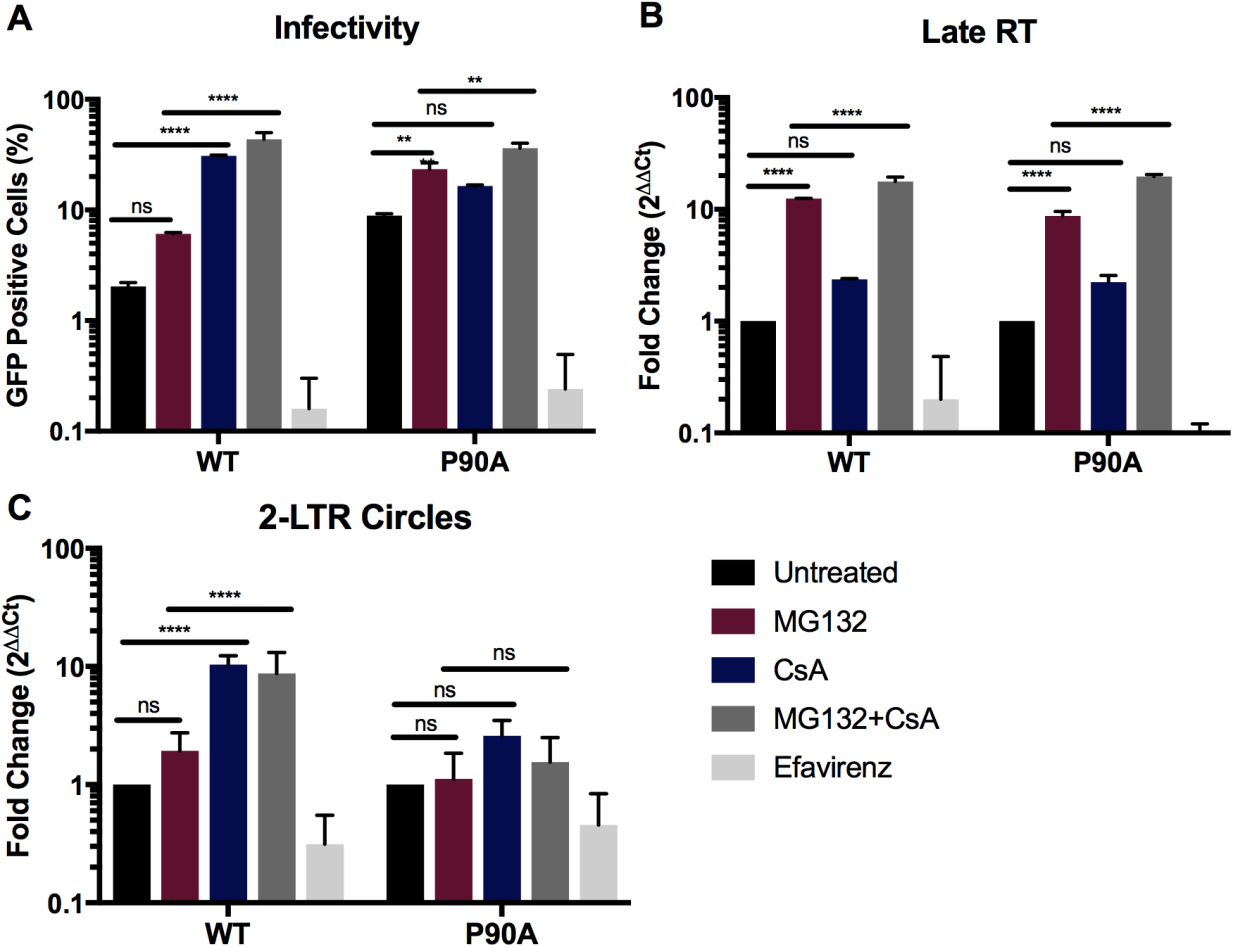
Proteasome inhibition does not relieve CsA sensitivity. Vero cells were infected with wild type or P90A HIV-1 reporter viruses and assayed for (A) infection, (B) late RT DNA synthesis or (C) 2-LTR circle formation. Infection was carried out in the presence of CsA, MG132 or both as indicated. Results shown are the mean values from 3 independent experiments; error bars reflect the standard error of the mean. Two-way ANOVA with Bonferroni post test * = p<0.05, ** = p<0.005, *** = p<0.0005, **** = p <0.0001.

We next asked how CypA promotes TRIM5α restriction at a step following binding to the viral capsid. A hallmark of TRIM5α restriction of retroviruses is the inhibition of reverse transcription. Previous studies demonstrated that proteasome activity is necessary for TRIM5α to inhibit reverse transcription but not for inhibition of infection. To ask whether proteasome activity is required for CypA-dependent inhibition of infection, we assayed HIV-1 infection in Vero cells treated with the proteasome inhibitor MG132, with CsA, and with both compounds. MG132 treatment alone only moderately enhanced infection of both wild type HIV-1 and the P90A mutant (Fig 5A). This effect was also observed in cells depleted of TRIM5α and CypA (S3 Fig). MG132 did not affect the extent to which CsA treatment stimulated infection of wild type HIV-1 (Fig 5A), indicating that proteasome activity is dispensable for HIV-1 inhibition by CypA. To confirm functional inhibition of the proteasome by MG132, we measured the accumulation of late reverse transcription products in these cells (Fig 5B). As a control for contamination of the virus with plasmid DNA, we included a parallel infection of Vero cells treated with Efavirenz, an inhibitor of HIV-1 reverse transcriptase. As expected, MG132 increased the levels of both WT and P90A reverse transcription products, indicating that proteasome activity was inhibited [14]. CsA treatment increased the accumulation of both WT or P90A reverse transcripts less than 2-fold in MG132-treated and untreated cultures (Fig 5B). These data indicate that proteasome activity is dispensable for CypA-dependent inhibition of infection, and are in agreement with a previous study reporting that CypA has a limited effect on TRIM5α inhibition of reverse transcription[36].

In the absence of proteasome activity, TRIM5α blocks HIV-1 infection by preventing its entry into the nucleus [14,52]. To test the hypothesis that CypA affects nuclear import in Vero cells, we monitored 2-LTR circle formation, both when reverse transcription was rescued by proteasome inhibition, and in the absence of proteasome inhibitor. 2-LTR circles are a product of host DNA repair mechanisms, and are commonly used as markers for HIV-1 nuclear import as they are selectively formed in the nucleus [3,53]. We observed a 10.3-fold increase in accumulation of 2-LTR circles in cells infected with wild type HIV-1 in the presence of CsA. By contrast, CsA enhanced the accumulation of 2-LTR circles formed by the P90A mutant by approximately 2-fold (Fig 5C). When proteasome activity was blocked by MG132 treatment, 2-LTR circle formation was moderately increased for the wild type virus (2.9 fold). The addition of MG132 and CsA together enhanced wild type 2-LTR circle formation by 8.6 fold, indicating that CypA inhibits 2-LTR circle formation in both the presence and absence of proteasome activity. Together, these observations suggest that CypA and TRIM5α cooperate to inhibit HIV-1 nuclear import and that this effect does not require proteasome activity.

### Selected HIV-1 CA mutants escape CypA-dependent restriction

**Fig 6:**
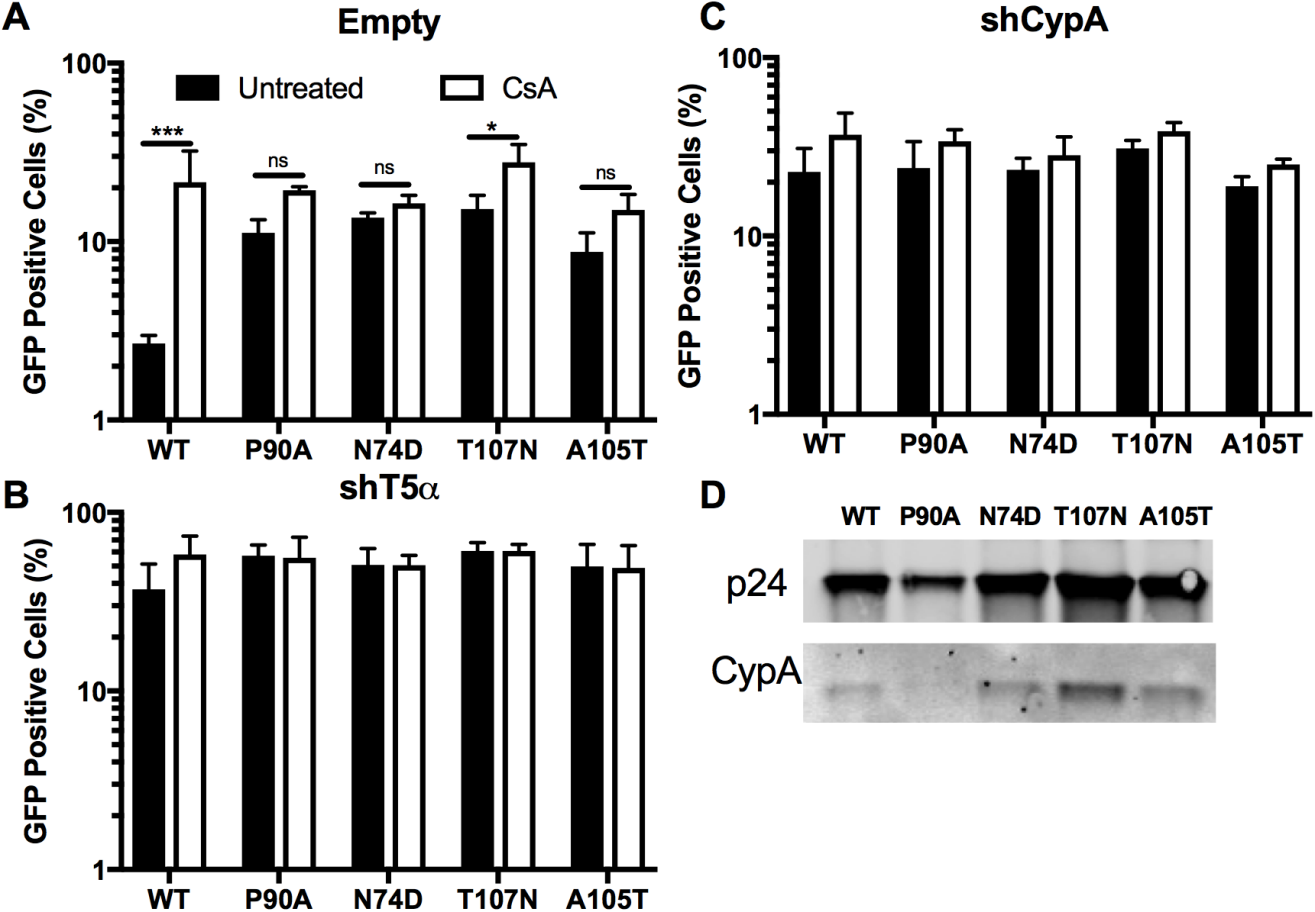
Selected CA mutants escape CsA-dependent TRIM5α restriction. Vero cell lines were assayed for infection by wild type HIV-1 and indicated CA mutants in the presence or absence of CsA. (A) Vero-shEmpty, (B) Vero-shT5α, or (C) Vero-shCypA were inoculated with WT or mutant HIV-GFP viruses. (D) Incorporation of CypA into HIV-1 virions. Virions were pelleted and analyzed by immunoblotting for CA protein and CypA. Values shown are mean values from 3 independent experiments; error bars represent the standard error of the mean. Two-way ANOVA with Bonferroni post test * = p<0.05, ** = p<0.005, *** = p<0.0005, ns = not significant.

Since we observed inhibition of 2-LTR circle accumulation by CypA, we next considered the mechanism by which CypA might inhibit nuclear import. CypA is known to alter the dependence of HIV-1 infection on several nuclear pore complex (NPC) associated host proteins thought to regulate entry into the nucleus ([28] and reviewed in [54]). Viral dependence on these NPC proteins has been mapped to a conserved region within HIV-1 CA known as the CPSF6-binding interface [55]. Several substitutions within this interface (N74D, A105T, and T107N) reduce the dependence of HIV-1 on one or more of these proteins, but maintain normal levels of infectivity [28,30,56–58]. To determine whether these residues are important for CypA-dependent restriction, we assayed the ability of CsA to stimulate infection by the N74D, A105T, and T107N mutant viruses in Vero cells. We found that CsA enhanced infection by these mutants by 2-fold or less, similar to the extent observed during infection with P90A HIV-1 (Fig 6A). To confirm that these mutants are restricted by TRIM5α, we repeated the infection in the presence and absence of CsA in Vero cells depleted of TRIM5α or CypA. We observed 5-fold or greater increase in infection by the mutants in cells depleted of TRIM5α, indicating that the mutants are restricted by TRIM5α despite the apparent resistance to the CypA-dependent component of restriction (Fig 6B). As previously observed with P90A HIV-1 and N-MLV, CypA depletion also slightly increased infection by the other CA mutants. (Fig 6C). To confirm that the mutants are able to bind CypA, we assessed incorporation of CypA into mutant HIV-1 particles by SDS-PAGE. CypA was detected in wild type and mutant virions, with the exception of the P90A mutant (Fig 6D). These results indicate that residues at the conserved CPSF6-binding interface in CA influence HIV-1 sensitivity to CypA-dependent TRIM5α restriction. These results also suggest that CypA binding is necessary but not sufficient to confer sensitivity to CypA-dependent TRIM5α restriction.

## Discussion

The host protein CypA can either increase or decrease the efficiency of HIV-1 infection [59–61]. In this study, we sought to determine the mechanism by which CypA promotes TRIM5α restriction of HIV-1 HIV-1 infection. We completed these studies in Vero cells, an African green monkey cell line exhibiting potent TRIM5α restriction. We observed that inhibition of either CypA or TRIM5α expression reduces restriction and the ability of CsA to stimulate HIV-1 infection (Fig 1). Using RNAseq, we provide evidence against expression of an endogenous TRIMCyp protein in the Vero cell line (Fig 2 and S2 Fig). Our data confirm that CypA inhibits HIV-1 infection by potentiating TRIM5α restriction of the virus.

TRIM5α inhibition of HIV-1 infection requires its binding to the capsid [62]. We provide two complementary forms of evidence that CypA does not act by promoting binding of TRIM5α to the viral capsid. First, we observed that TRIM5α binding to capsid-like CA tubes *in vitro* is not affected by CypA (Fig 3). Second, we showed that CypA binding to the incoming viral capsid did not alter the ability of HIV-1 particles to saturate TRIM5α-dependent restriction *in trans* (Fig 4). These results suggest that CypA affects TRIM5α restriction at a step after its binding to the capsid. Our observation that inhibition of CypA increased the levels of 2-LTR circles, but not total reverse transcripts confirmed this hypothesis (Fig 5B and 5C, [36]). Accordingly, inhibition of cellular proteasome activity did not alter the ability of CypA to potentiate TRIM5α restriction, consistent with an effect mediated after reverse transcription (Fig 5A). Previous reports have suggested that the ability of TRIM5α to block nuclear import of HIV-1 is revealed only upon the inhibition of proteasome activity [14,52,63]. As CsA treatment rescues nuclear import in the presence and absence of proteasome activity, we suggest that inhibition of nuclear import is a distinct step in TRIM5α restriction of HIV-1, and depends on CypA.

How might CypA and TRIM5α cooperate to inhibit HIV-1 nuclear entry? In permissive human cells CypA promotes nuclear entry of HIV-1 [28,60]. Depletion of CypA, or mutation of the CA binding site, reduces HIV-1 dependence on specific components of the nuclear pore complex (NPC) [28–30,57,64]. We observed that CsA did not stimulate infection of Vero cells by CA mutants exhibiting reduced dependence on canonical HIV-1 NPC components, yet the mutants were sensitive to TRIM5α restriction and retained CypA binding (Fig 6). Therefore, we hypothesize that NPC proteins binding to this region of HIV-1 CA also contribute to CypA-dependent TRIM5α restriction. Based on these observations, we propose that CypA directs HIV-1 to engage the NPC in manner that promotes TRIM5α restriction. Perhaps CypA enhances restriction by stabilizing the capsid and delaying a nuclear uncoating step, thereby increasing the time of cytoplasmic residency. Alternatively, capsid-associated TRIM5α could sterically hinder HIV-1 capsid interactions with CypA-dependent NPC cofactors in order to block nuclear import. Although the actual mechanism remains to be determined, this model may also explain the potentiating effects of CypA observed with other capsid-targeting inhibitors, including the host restriction factor MxB and the small molecule antiviral compound PF74 [32,33,65,66]. The same CA mutations that permit escape from CypA-dependent TRIM5α restriction, also partially rescue restriction mediated by MxB and by PF74, suggesting that CypA uses a common mechanism involving interactions of the virus with nuclear pore components to potentiate restriction of HIV-1 infection by a variety of protein and small molecule inhibitors.

## Supporting information

S1 Fig Quantification of target gene mRNA following shRNA mediated depletion. Total RNA from puromycin selected populations of Vero cells transduced with pLKO.1 vectors containing shRNA directed against TRIM5α or CypA was subjected to RT-qPCR to quantify mRNA transcripts present 48hrs after selection.
(TIFF)

S2 Fig Track view read mapping for each Vero and OMK samples to OMK TRIMCyp. **A**. and **C.** Alignment of reads across a 400bp section of OMK TRIMCyp containing the TRIM and CypA homology junction. Green and red lines indicate forward and reverse reads. Multicolored blocks indicate mismatches with the main sequence. Faded red or faded green portions indicate regions of the read which do not align to reference sequence. Pink arrows denote the junction site and the beginning of the CypA homology region. **B** and **D** Graphical representation of read coverage across the junction region.
(PDF)

S3 Fig Proteasome sensitivity in Vero cells depleted of TRIM5α and CypA. A) Vero cells depleted of TRIM5 (shT5α) or CypA (shCypA), and control (Empty) were assayed for infection by WT or P90A HIV-1 reporter viruses, in the presence and absence of CsA. Results shown are the mean values from 3 independent experiments, error bars reflect the standard error of the mean.
(TIFF)

S1 Table *De novo* assembly and mapping statistics of Vero and OMK transcriptomes. (PDF)

S2 Table OMK TRIMCyp BLAST summary statistics. (PDF)

S3 Table Mapping statistics for OMK TRIMCyp. (PDF)

S4 Table Absolute concentration and titer of HIV-1 Viruses. (PDF)
