# Supplementary figures and images for: Cyclophilin A potentiates TRIM5α inhibition of HIV-1 nuclear import without promoting TRIM5α binding to the viral capsid
Source: PLoS One. 2017 Aug 2;12(8):e0182298. doi: 10.1371/journal.pone.0182298 (PMC5540582; doi:10.1371/journal.pone.0182298)

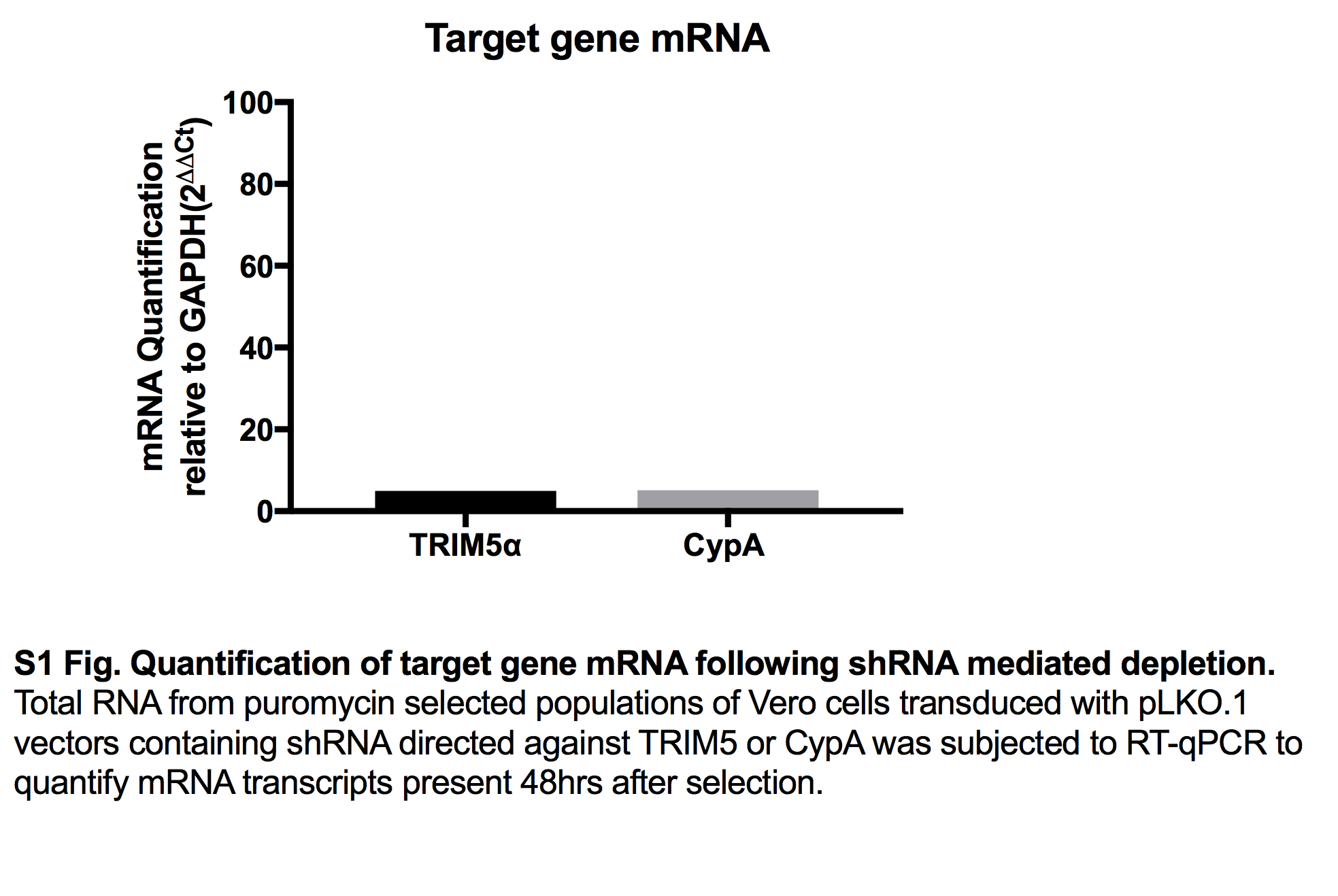

Supplement: S1 Fig — Total RNA from puromycin selected populations of Vero cells transduced with pLKO.1 vectors containing shRNA directed against TRIM5α or CypA was subjected to RT-qPCR to quantify mRNA transcripts present 48hrs after selection. (TIFF) [file pone.0182298.s001.tiff]

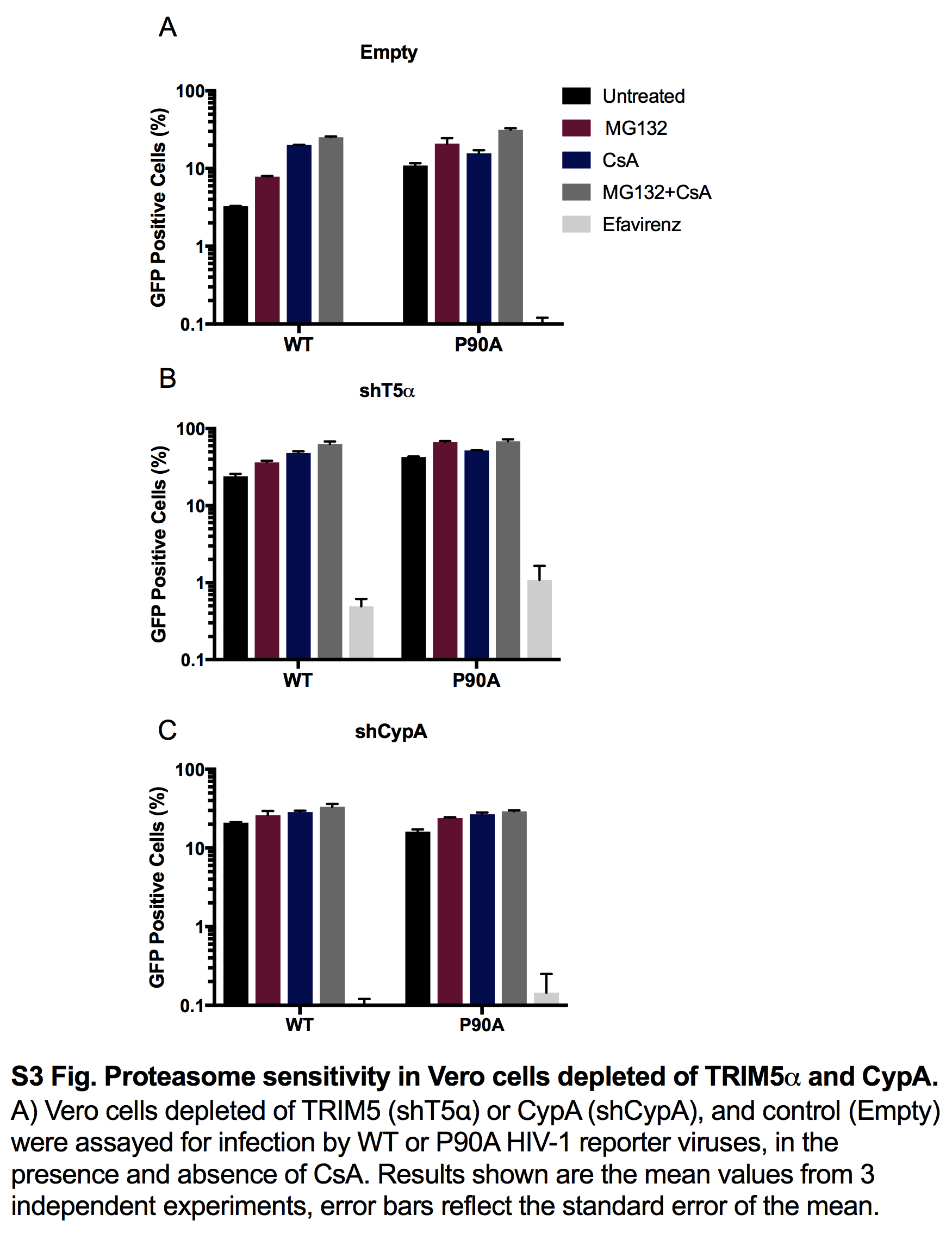

Supplement: S3 Fig — A) Vero cells depleted of TRIM5 (shT5α) or CypA (shCypA), and control (Empty) were assayed for infection by WT or P90A HIV-1 reporter viruses, in the presence and absence of CsA. Results shown are the mean values from 3 independent experiments, error bars reflect the standard error of the mean. (TIFF) [file pone.0182298.s003.tiff]
