# Supplementary material for: Cyclophilin A potentiates TRIM5α inhibition of HIV-1 nuclear import without promoting TRIM5α binding to the viral capsid
Source: PLoS One. 2017 Aug 2;12(8):e0182298. doi: 10.1371/journal.pone.0182298 (PMC5540582; doi:10.1371/journal.pone.0182298)
Supplement: S2 Fig — A. and C. Alignment of reads across a 400bp section of OMK TRIMCyp containing the TRIM and CypA homology junction. Green and red lines indicate forward and reverse reads. Multicolored blocks indicate mismatches with the main sequence. Faded red or faded green portions indicate regions of the read which do not align to reference sequence. Pink arrows denote the junction site and the beginning of the CypA homology region. B and D Graphical representation of read coverage across the junction region. (PDF) [file pone.0182298.s002.pdf]

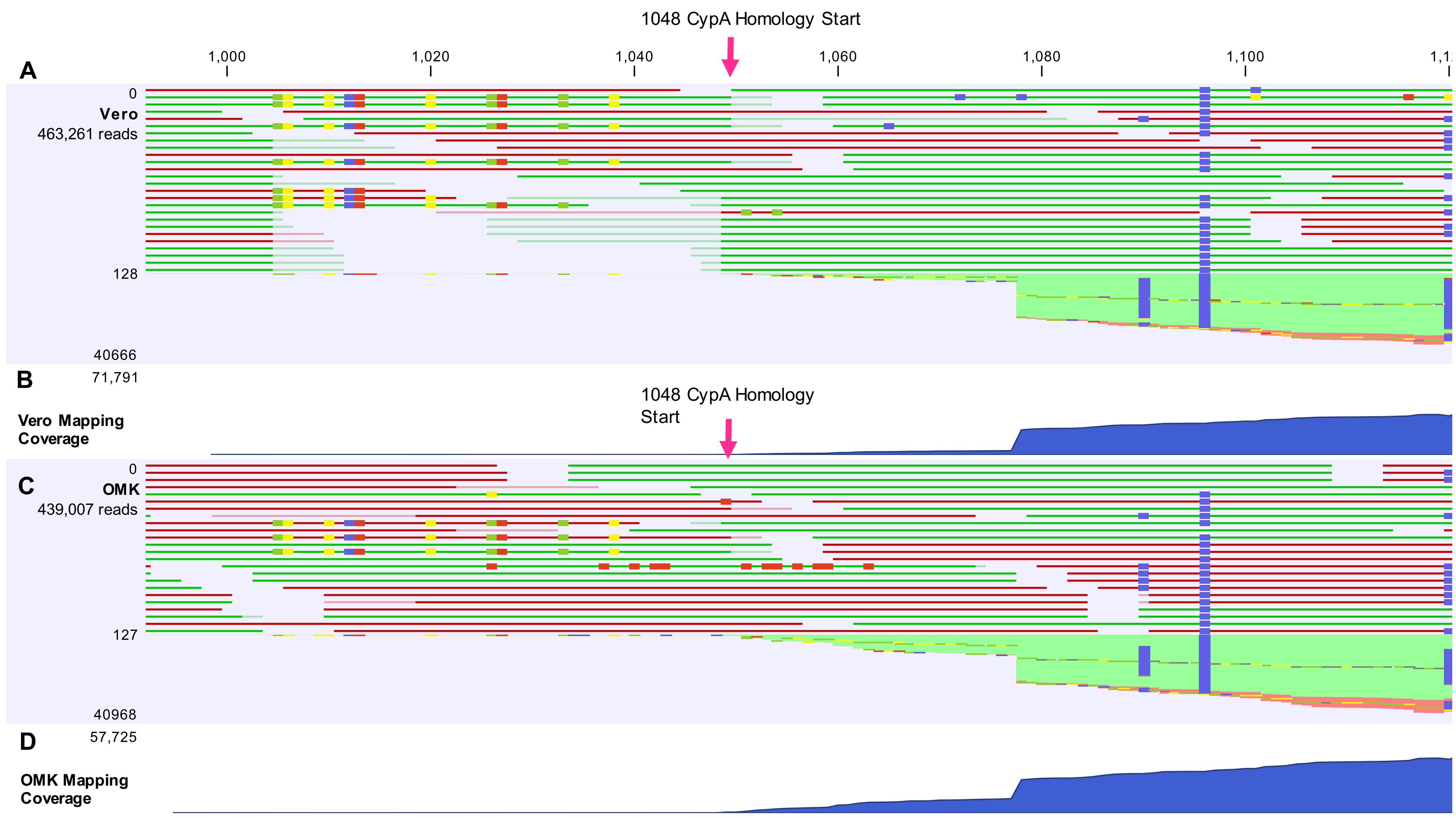

S2 Fig. Track view read mapping for each Vero and OMK samples to OMK TRIMCyp. A. and **C.** Alignment of reads across a 400bp section of OMK TRIMCyp containing the TRIM and CypA homology junction. Green and red lines indicate forward and reverse reads. Multicolored blocks indicate mismatches with the main sequence. Faded red or faded green portions indicate regions of the read which do not align to reference sequence. Pink arrows denote the junction site and the beginning of the CypA homology region. **B** and **D** Graphical representation of read coverage across the junction region
