# Supplementary material for: Cyclophilin A potentiates TRIM5α inhibition of HIV-1 nuclear import without promoting TRIM5α binding to the viral capsid
Source: PLoS One. 2017 Aug 2;12(8):e0182298. doi: 10.1371/journal.pone.0182298 (PMC5540582; doi:10.1371/journal.pone.0182298)
Supplement: S1 Table — (PDF) [file pone.0182298.s004.pdf]

Table 1.1 ***De Novo Assembly and Mapping Statistics of Vero and OMK Transcriptomes***

	Samples	Total number of reads	% Mapped Reads	Total number of contigs	Longest contig length (bp)
	1	78,022,554	64.06	14,030	14,986
**OMK**	2	70,520,226	62.992	12,638	13,931
	3	56,613,962	60.466	11,126	13,692
	1	77,766,242	78.951	10,473	19,578
**Vero**	2	73,627,466	75.059	11,408	17,631
	3	69,573,622	73.425	12,430	19,555
