# Supplementary material for: Cyclophilin A potentiates TRIM5α inhibition of HIV-1 nuclear import without promoting TRIM5α binding to the viral capsid
Source: PLoS One. 2017 Aug 2;12(8):e0182298. doi: 10.1371/journal.pone.0182298 (PMC5540582; doi:10.1371/journal.pone.0182298)
Supplement: S2 Table — (PDF) [file pone.0182298.s005.pdf]

Table 1.2 OMK TRIMCyp BLAST Summary Statistics

	Aligned to OMK TRIMCyp	Aligned to Junction	E Value	% Query Overlap	% Identity
OMK_Contig_150	Yes	Yes	0	94.4	98.6
OMK_Contig_276	Yes	Yes	0	79	99.6
Vero_Contig_236	Yes	No	0	40.7	94.3
Vero_Contig_364	Yes	No	0	40.8	94.3
