# Supplementary material for: Cyclophilin A potentiates TRIM5α inhibition of HIV-1 nuclear import without promoting TRIM5α binding to the viral capsid
Source: PLoS One. 2017 Aug 2;12(8):e0182298. doi: 10.1371/journal.pone.0182298 (PMC5540582; doi:10.1371/journal.pone.0182298)
Supplement: S3 Table — (PDF) [file pone.0182298.s006.pdf]

Table 1.3 Mapping Statistics for OMK TRIMCyp

	Total mapped reads	% Total Reads	Reads in Pairs	Broken Paired Reads
Vero	264,747	0.12%	120,348	144,399
OMK	292,061	0.14%	175,720	116,341
