# Supplementary material for: Cyclophilin A potentiates TRIM5α inhibition of HIV-1 nuclear import without promoting TRIM5α binding to the viral capsid
Source: PLoS One. 2017 Aug 2;12(8):e0182298. doi: 10.1371/journal.pone.0182298 (PMC5540582; doi:10.1371/journal.pone.0182298)
Supplement: S4 Table — (PDF) [file pone.0182298.s007.pdf]

Table S4. Titer of HIV-1 Viruses

	CRFK titer (i.u/μl)	p24 (ng/ml)
Wildtype	503	1489
P90A	1265	2575
N74D	1267	2471
T105N	2083	3522
A105T	986	2742
